# Nucleocapsid protein-based vaccine provides protection in mice against lethal Crimean-Congo hemorrhagic fever virus challenge

**DOI:** 10.1371/journal.pntd.0006628

**Published:** 2018-07-16

**Authors:** Marko Zivcec, David Safronetz, Dana P. Scott, Shelly Robertson, Heinz Feldmann

**Affiliations:** 1 Department of Medical Microbiology and Infectious Diseases, University of Manitoba, Winnipeg, Manitoba, Canada; 2 Laboratory of Virology, Division of Intramural Research, National Institute of Allergy and Infectious Disease, National Institutes of Health, Hamilton, Montana, United States of America; 3 Rocky Mountain Veterinary Branch, Division of Intramural Research, National Institute of Allergy and Infectious Disease, National Institutes of Health, Hamilton, Montana, United States of America; University of Texas Medical Branch, UNITED STATES

## Abstract

Crimean-Congo hemorrhagic fever (CCHF) is an acute, often fatal viral disease characterized by rapid onset of febrile symptoms followed by hemorrhagic manifestations. The etiologic agent, CCHF orthonairovirus (CCHFV), can infect several mammals in nature but only seems to cause clinical disease in humans. Over the past two decades there has been an increase in total number of CCHF case reports, including imported CCHF patients, and an expansion of CCHF endemic areas. Despite its increased public health burden there are currently no licensed vaccines or treatments to prevent CCHF. We here report the development and assessment of the protective efficacy of an adenovirus (Ad)-based vaccine expressing the nucleocapsid protein (N) of CCHFV (Ad-N) in a lethal immunocompromised mouse model of CCHF. The results show that Ad-N can protect mice from CCHF mortality and that this platform should be considered for future CCHFV vaccine strategies.

## Author summary

Crimean-Congo hemorrhagic fever (CCHF) is a tick-borne disease that can manifest as a viral hemorrhagic fever syndrome. The CCHF virus is widely spread throughout the African continent, the Balkans, the Middle East, Southern Russia and Western Asia where it remains a serious public health concern. Currently, there are no licensed treatments or vaccines available, and medical countermeasures are urgently needed. We developed an adenovirus vector vaccine based on the conserved structural nucleoprotein (N) as the antigen. A prime-boost approach showed promising efficacy in the most widely used immunocompromised mouse model. This vaccine approach demonstrates a role for N in protection and suggests its consideration for future CCHFV vaccine strategies.

## Introduction

Crimean-Congo hemorrhagic fever (CCHF) is an acute infectious disease with a wide geographic distribution and an average case fatality rate of approximately 20–30% [1, 2]. The etiological agent, CCHF orthonairovirus (CCHFV), belongs to the *Orthonairovirus* genus of the *Nairoviridae* family. The CCHFV genome consists of tri-segmented, negative-sense RNA referred to as the small (S), medium (M) and large (L) segments encoding the nucleocapsid protein (N), the glycoprotein precursor (GPC) and the viral RNA-dependent-RNA-polymerase (L), respectively [2, 3].

CCHFV is primarily maintained in and transmitted by ticks in the *Hyalomma* genus of the *Ixodidae* family [2]. The virus has a wide host range and causes a transient viremia in many wild, domesticated and laboratory mammals [1, 4, 5]. Humans usually acquire infection by tick bite or through unprotected contact with body fluids of infected animals or humans; additionally, several nosocomial outbreaks have been reported [1, 2]. In contrast to humans, adult immuno-competent mammals have not yet been reported to develop signs of disease [1, 2, 6]. This has impaired animal model development and hampered the testing of medical countermeasures against CCHF. CCHFV is an interferon-sensitive virus and its replication is highly reduced by treatment with interferon in interferon-signaling competent cells [7–9]. These observations led to the discovery that adult mice with gene knockouts in interferon signaling pathways, such as the signal transducer and activator of transcription-1 (STAT1^-/-^) and the interferon α/β receptor (IFNAR^-/-^) mouse strains, are highly susceptible to CCHFV infection mimicking some hallmarks of human disease [10–12].

CCHF is considered endemic in more than 30 countries throughout the African continent, the Balkans, the Middle East, Southern Russia and Western Asia [1, 4]. Over the past 20 years, CCHF has emerged or re-emerged in several countries often with dramatically increased case numbers. Furthermore, there has been a marked increase of imported cases of CCHF in European and South Asian countries [13–17]. The root causes of the increased CCHF incidence rates are not fully understood, but factors such as shifts in climate patterns, animal and human migratory patterns, population increase in livestock and wildlife accompanied by changes in agricultural practices and increased land use may be responsible [18, 19]. Thus, there is a pressing need to develop prophylactic and therapeutic countermeasures against this significant emerging zoonosis.

Over the past few years several vaccine candidates have been evaluated for protective efficacy in pre-clinical studies. Among those were vaccines based on DNA, viral subunits, whole inactivated virus, virus-like particle (VLP) and viral vectors such as modified vaccinia virus Ankara (MVA) [20–25]. Full protection against lethal CCHFV challenge in a mouse model was achieved with DNA plasmids expressing GPC subunits and N, as well as a MVA vector expressing the GPC open reading frame. Partial protection was observed with whole inactivated virus preparations, a DNA plasmid expressing GPC, VLPs (consisting of N, GPC and L), and a combined plasmid DNA (GPC subunits and N)/VLP vaccination approach [23–25]. No protection was achieved with a soluble glycoprotein subunit vaccine and MVA expressing CCHFV N. Overall, these studies implicate immune responses to the glycoproteins as most important for protection, however the glycoprotein subunit platform has not shown any protection despite inducing a significant antibody response [22]. Due to the high genetic diversity of the surface glycoproteins (69–99% amino acid identity between CCHFV strains), a broadly efficacious vaccine may need an additional, more genetically conserved CCHFV antigen such as N (91–99% amino acid identity between CCHFV strains) [26, 27].

Here we constructed and characterized an experimental CCHF vaccine vector based on human adenovirus type 5 (Ad) expressing the CCHFV N (Ad-N). Following infection with the recombinant Ad-N the CCHFV N protein was detected in cell lysates. Mice immunized with Ad-N developed an anti-N humoral immune response. A single dose of Ad-N resulted in 30% protection of IFNAR^-/-^ mice against lethal CCHFV challenge. This could be further improved by a prime-boost regimen to 78% protection. These results indicate a significant role of N as a protective component of a CCHFV vaccine.

## Materials and methods

### Ethics and biosafety statements

Animal experiments were approved by the Institutional Animal Care and Use Committee of the Rocky Mountain Laboratories (RML) and were performed following the guidelines of the Association for Assessment and Accreditation of Laboratory Animal Care, International (AAALAC) by certified staff in an AAALAC approved facility (#A4149-01). All procedures involving infectious CCHFV were performed in the RML Biosafety Level (BSL) 4 facility and all standard operating procedures (SOPs) including sample inactivation were approved by the Institutional Biosafety Committee (IBC).

### Challenge virus

CCHFV Strain IbAr 10200 (kindly provided by the University of Texas Medical Branch, Galveston, TX, USA; at that time Michael Holbrook), was propagated in Scott and White No. 13 (SW13) cells maintained in Leibovitz’s L-15 medium (both from ATCC) supplemented with 10% heat-inactivated fetal bovine serum (FBS), 100 mM L-glutamine, and 50 U/mL penicillin, 50 μg/mL Streptomycin (Sigma-Aldrich) in an environment not enriched in CO_2_.

### Vaccine vector

The adenovirus (Ad) expressing the complete open reading frame (ORF) of the N of CCHFV Strain IbAr 10200 (Ad-N) (NCBI Ref seq U88410.1 nucleotide 56–1504) or wild type Ad (Ad-wt) were constructed and rescued using the Adeno-X Adenoviral System 3 and titered using the Adeno-X Rapid Titer Kit according to manufacturer’s instructions (both from Clontech). Ad were propagated in 293 cells (ATCC) maintained in Dulbecco’s modified Eagle medium (DMEM) supplemented with 10% FBS, 100 mM L-glutamine, and 50 U/mL penicillin, 50 μg/mL Streptomycin (obtained from Sigma-Aldrich).

### Immunoblot analysis

293 cells were infected with Ad-wt and Ad-N with a multiplicity of infection (MOI) of ~5. Cell lysates were harvested (scraped into PBS-Tween 0.05% [PBST]) on day 2 post infection for immunoblot analysis. For SDS-PAGE, samples were mixed with 2× SDS-PAGE loading buffer (1:1, v/v), boiled for 10 minutes, centrifuged and applied to 10% SDS-PAGE. Proteins were blotted onto a nylon membrane (GE healthcare) followed by blocking overnight in 5% milk-PBST solution at 4 C°. The membrane was incubated with a rabbit anti-N (N_1028_) peptide polyclonal serum (Thermo Fisher Scientific Inc.) [12] followed by a goat anti-rabbit HRP conjugated antiserum. Detection was performed using Pierce ECL Plus Western Blotting Substrate (both from Thermo Fisher Scientific Inc.) according to the manufacturer's protocol.

### Vaccine efficacy studies

IFNAR^-/-^ mice (6 to 12-week-old; on C57BL/6 background) were obtained from an in-house breeding colony. Groups of IFNAR^-/-^ mice (n = ranging from 3–18 animals per group) were vaccinated with 1.25×10^7^ infectious units (IFU) of Ad-wt or Ad-N by the intramuscular route delivered into the hind-leg musculature (day -28 for prime-only and day -56 for prime/boost group; 50 μL total volume). IFNAR^-/-^ mice in prime/boost experiments were boosted 4 weeks (day -28) post vaccination with 10^8^ IFU of the homologous Ad construct by the intranasal route (25 μL per nostril). Mice were boosted by the intranasal route as this vaccine regimen has previously been shown to bypass pre-existing Ad vector immunity and stimulate a more robust immune response against the Ad encoded antigens [28]. Vaccinated mice were acclimatized to the BSL4 environment for 5–7 days prior to CCHFV challenge. Four weeks after vaccination or boost (day 0) IFNAR^-/-^ mice were challenged with 1000 LD_50_ (50 TCID_50_) of CCHFV by the subcutaneous route delivered to the intrascapular region (50 μL total volume). Animals were monitored daily for clinical signs with group weights being recorded. On day 3 post infection with CCHFV, 9 mice from the distinct groups were exsanguinated by cardiac puncture under anesthesia with whole blood collected into EDTA tubes (BD Biosciences) for RNA extraction and frozen at −80°C for virus isolation. Liver and spleen specimens were collected for pathological evaluation or immediately frozen at -80°C for virus isolation. The remaining mice (n = 3, 6 or 9) per vaccine group were monitored daily for 30 days post infection.

### Enzyme-linked immunosorbent assay (ELISA)

For antibody (IgG) detection we used an ELISA based on whole CCHFV particle antigen. Supernatants from CCHFV infected (positive antigen) and mock-infected (negative antigen) SW13 cells were harvested, cleared from cell debris through low-speed centrifugation, diluted 1:200 in 0.05% PBST and treated by gamma-irradiation (8 MRads). Maxisorp plates (96-well; Thermo Fisher Scientific Inc.) were coated with positive and negative antigen in 5% skim milk in PBST overnight at 4°C. Mouse serum was serially diluted two-fold starting at a dilution of 1:50, added to the plates and incubated for 1 hour at room temperature. Detection was performed using a goat anti-mouse peroxidase-conjugated IgG (KPL) at a 1:1000 dilution followed by treatment with ABTS peroxidase substrate system (KPL) as per manufacturer’s instructions. The cut-off was set at >3 standard deviations above the reading of negative samples. The data are reported as inverse dilutions.

### Detection of viral genomic RNA

CCHFV S segment specific quantitative RT-PCR and tissue titration were carried out as previously described [12].

### Histopathology and immunohistochemistry (IHC)

Tissue samples were treated and fixed in 10% formalin according to approved standard operating procedure. Fixed samples were processed and either stained with hematoxylin and eosin (H&E) or N_1028_ polyclonal antiserum for histopathology or IHC, respectively, as described previously [12]. Slides were examined by a veterinary pathologist and scored as follows: 0 = no obvious pathological changes; 1 = minimal increase in the number of inflammatory cells and hepatocellular necrosis; 2 = mildly increased numbers of inflammatory cells, hepatocellular necrosis or lymphocytolysis; 3 = moderately increased numbers of inflammatory cells, and hepatocellular necrosis or lymphocytolysis; and 4 = highly increased numbers of inflammatory cells and multifocal hepatocellular necrosis or lymphocytolysis.

### Statistical analysis

All physiological parameters were compared and analyzed using one-way or two-way analysis of variance (ANOVA) with Dunnet’s posttest on GraphPad Prism v5.00 (GraphPad Software).

## Results

### Antigen expression and immunogenicity of Ad-based vaccine vector

Confluent 293 cells were infected with Ad-wt and Ad-N at a MOI of 5. Expression of CCHFV N was verified only in cell lysates from Ad-N-infected cells by immunoblot using an N-specific antiserum. (S1A Fig). To determine the immunogenicity of CCHFV N in an immunocompromised host, IFNAR^-/-^ mice (n = 3) were vaccinated intramuscularly with Ad-N (1.25×10^7^ IFU) followed by an intranasal boost (10^8^ IFU) four weeks later; seroconversion was assessed by IgG ELISA. All three mice developed detectable IgG antibodies responses to CCHFV N with titers ≥1:6400 (S1B Fig).

**Fig 1:**
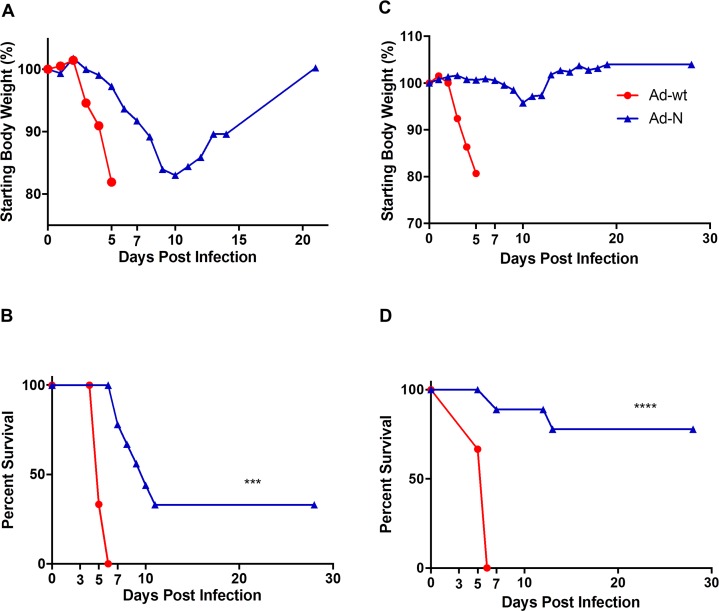
Efficacy of single-dose and prime-boost vaccination. (A,B) Single-dose vaccination. IFNAR^-/-^ mice (n = 9 for Ad-N; n = 6 for Ad-wt) were vaccinated with recombinant adenoviruses (1.25×10^7^ ifu) 28 days prior to lethal CCHFV infection (1000 LD_50_). The animals were monitored for weight as a group (A) and disease progression/survival (B) over 30 days. (C,D) Prime-boost vaccination. IFNAR^-/-^ mice (n = 9 for Ad-N; n = 3 for Ad-wt) were vaccinated with recombinant adenoviruses 56 (1.25×10^7^ IFU; intramuscular) and 28 (10^8^ IFU; intranasal) days prior to lethal CCHFV infection (1000 LD_50_). The animals were monitored for weight (C) and disease progression/survival (D) over 30 days. Data is reported as the means. *** p<0.001, ****p<0.0001.

### Efficacy of single-dose and prime-boost vaccination

IFNAR^-/-^ mice were vaccinated intramuscularly with the recombinant adenoviruses (1.25×10^7^ IFU) 28 days before CCHFV challenge (1000 LD_50_). Mice vaccinated with Ad-wt (n = 6) rapidly lost weight and succumbed to infection within 6 days (Fig 1A and 1B). Vaccination with Ad-N (n = 9) resulted in partial protection against lethal CCHFV challenge (33% survival, p>0.05) with reduced clinical signs (i.e. weight loss) and increased survival times (8.5 days vs 5 days survival, Ad-N vs Ad vaccinated, p<0.001) in those mice that succumbed to infection (Fig 1A and 1B).

A prime-boost strategy was employed next with the prime being administered intramuscularly and the boost intranasally, a strategy that has resulted in enhanced protective efficacy before [28]. For this, IFNAR^-/-^ mice were immunized at day -56 (1.25×10^7^ IFU) and boosted on day -28 (10^8^ IFU) with recombinant adenoviruses, challenged on day 0 with CCHFV (1000 LD_50_) and monitored for survival. As with the single-dose vaccination, Ad-wt vaccinated mice (n = 3) rapidly lost weight and succumbed to infection by day 6 post infection (Fig 1C and 1D). Ad-N vaccinated animals (n = 9) showed increased protection from lethal CCHFV challenge (78% survival, p<0.0001) with reduced clinical signs including weight loss and increased survival times in those mice that succumbed to infection (Fig 1C and 1D).

### CCHFV loads and antigen distribution after single-dose and prime-boost vaccinations

**Fig 2:**
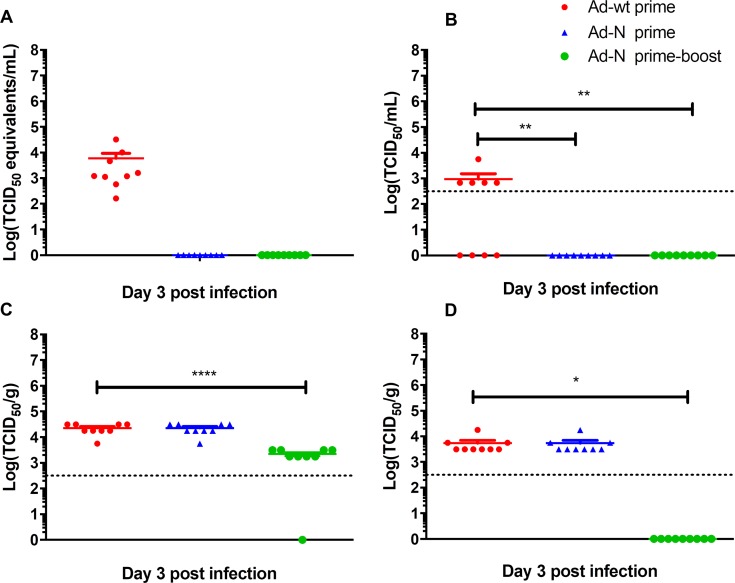
CCHFV loads in single-dose and prime-boost vaccinated and challenged mice. Groups of IFNAR^-/-^ mice were either single-dose (1.25×10^7^ IFU; intramuscular) or prime-boost (1.25×10^7^ IFU; intramuscular / 10^8^ IFU; intranasal) vaccinated with Ad-N or Ad-wt and challenged with 1000 LD_50_ of CCHFV 28 days following final vaccination. Mice (n = 9 per group) were anesthetized, bleed and euthanized to harvest organ samples on day 3 post CCHFV challenge. Viral loads were analyzed by quantitative RT-PCR or infectivity assay. (A) Viremia analyzed by quantitative RT-PCR; (B) Viremia analyzed by TCID_50_ assay; (C) liver virus load analyzed by TCID_50_ assay; (D) spleen viral loads analyzed by TCID_50_ assay. Data is shown as individual organ data points, the mean and the standard error of the mean. The dotted line illustrates the limit of detection of the TCID_50_ assay. * p<0.05, ** p<0.01, **** p<0.0001.

At day 3 post CCHFV challenge, mice (n = 9) were euthanized and their liver, spleen and blood were sampled for virus load titrations and histopathology. In contrast to Ad-wt vaccinated animals, IFNAR^-/-^ mice vaccinated with Ad-N either by a single-dose or a prime-boost approach did not show detectable viremia as analyzed by quantitative RT-PCR and virus infectivity assay (Fig 2A and 2B). Notably, liver and spleen viral loads on day 3 post infection of animals vaccinated with a single dose of Ad-N were similar to those detected in Ad-wt vaccinated animals (Fig 2C and 2D). In contrast, liver and spleen viral loads were significantly reduced following prime-boost vaccination with Ad-N. This observation was most prominent in the spleen for yet unknown reasons (Fig 2C and 2D).

**Fig 3:**
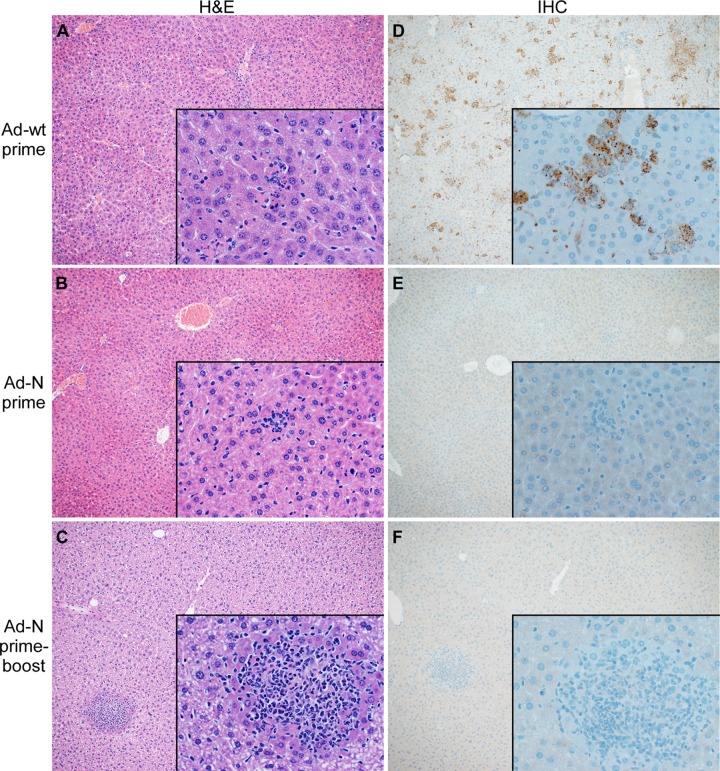
Liver histopathology and CCHFV antigen distribution in single-dose and prime-boost vaccinated and challenged mice. Groups of IFNAR^-/-^ mice were either single, (1.25×10^7^ IFU; intramuscular) or prime-boost (1.25×10^7^ IFU; intramuscular / 10^8^ IFU; intranasal) vaccinated with Ad-N or Ad-wt and challenged with 1000 LD_50_ of CCHFV 28 days following final vaccination. Mice (n = 9 per group) were anesthetized, bleed and euthanized to harvest organ samples on day 3 post CCHFV challenge. Thin-sections of liver material were stained with hematoxylin and eosin (H&E) or with N_1028_ rabbit polyclonal serum (anti-CCHFV N serum) (IHC). (A) Liver H&E of control-vaccinated mice (Ad-wt), (B) Liver H&E of prime-vaccinated mice (Ad-N); (C) Liver H&E of prime-boost-vaccinated mice (Ad-N); (D) Liver IHC of control-vaccinated mice (Ad-wt); (E) Liver IHC of prime-vaccinated mice (Ad-N); (F) Liver IHC of prime-boost-vaccinated mice (Ad-N). Images are at a magnification of 10x with 500x insets.

Ad-wt vaccinated mice developed multifocal to coalescing hepatocellular necrosis with infiltration of viable and degenerate neutrophils in the liver (Fig 3A), and the spleens demonstrated mild to marked acute necrotizing splenitis with loss of lymphocytes (S2A Fig) as shown previously for CCHFV-infected IFNAR^-/-^ mice [12]. IFNAR^-/-^ mice vaccinated with a single dose of Ad-N had less severe hepatic lesions compared to Ad-wt vaccinated animals consisting of mild focal necrosis and infiltration of small numbers of viable and degenerate neutrophils (Fig 3B). There were no splenic lesions detectable (S2B Fig). This result was improved by prime-boost vaccination which resulted in further reduction of hepatic lesions (Fig 3C) and absence of splenic lesions (S2C Fig). IHC demonstrated high amounts of CCHFV N antigen in the liver and spleen of those animals immunized with Ad-wt vector (Fig 3D; S2D Fig), whereas mice immunized with Ad-N showed strongly reduced numbers of CCHFV antigen-positive cells in liver and spleen with the lowest numbers for those animals vaccinated with the prime-boost regimen (Fig 3E and 3F; S2E and S2F Fig). Antigen-positivity was scattered throughout the liver and spleen and was associated with cells morphologically consistent with hepatocytes, Kupffer cells, macrophages and endothelial cells, as previously reported [12].

## Discussion

Due to high case fatality rates, potential of human-to-human transmission, increasing likelihood of imported cases and expanding endemic region, CCHFV is a serious threat to public health. This has been recognized by the World Health Organization, which added CCHFV to the list of priority pathogens (http://www.who.int/blueprint/priority-diseases/en/). Therefore, development and evaluation of countermeasures, especially vaccines, is of critical importance in mitigating the detrimental impact of CCHF.

The only CCHF disease models are certain immunocompromised and/or humanized mouse strains [2]. The mouse strains include the interferon signaling deficient IFNAR^-/-^ and STAT1^-/-^ mouse strains and the humanized Hu-NSG-SGM3 mice [10–12, 29]. The Hu-NSG-SGM3 express certain human cytokines and human leukocytes, however the disease progression is somewhat atypical as the disease is primarily neurological [29]. Both the STAT1^-/-^ and IFNAR^-/-^ mice possess complete sets of murine immune systems, but their cells either have altered response to interferon signaling or do not respond to type I interferon signaling, respectively, leading to rapid disease more reminiscent of human hemorrhagic fever [10–12]. IFNAR^-/-^ mice are highly susceptible to severe disease caused by several viral agents [11, 12, 30–31] due to lower and/or altered immune responses to infections compared with wild type mice [32–35]. The IFNAR^-/-^ mice frequently do not respond as quickly to infection as wild type mice and, furthermore, CCHFV replicates to higher levels than in wild type mice [12]. The increased difficulty in protecting IFNAR^-/-^ mice from infection seems associated with insufficient immune responses due to inadequate cross-priming of antigen presenting cells [32]. Thus, efficacy testing of vaccines in this model can be difficult as protective vaccines must elicit proper immune responses by circumventing IFNAR^-/-^ dampened antigen priming, and must stimulate an effective adaptive immune response that can compensate for the lack of antiviral state activation and reduction of CCHFV replication normally mediated by type I interferon signaling. Therefore, IFNAR^-/-^ mice should be considered a “higher bar” for efficacy testing of vaccines than fully immunocompetent rodent models.

CCHFV vaccine development is further complicated by limited information on both B- and T-cell epitope requirements for the development of an effective immune response; and the type(s) of immune responses necessary for protection from disease. The MVA, plasmid DNA and VLP vaccine platforms have had success in protecting mice from lethal CCHFV challenge, and the protection afforded was dependent on both humoral and cell-mediated immunity [20, 21, 24, 25, 36]. In addition, all the platforms strongly suggests that immune responses directed against the GPC, whether antibody or T cell driven, are essential for protection in rodents [20–25, 36]. While vaccination regimens that focus on a single antigen have been successful, an ideal vaccination candidate would facilitate immune response against multiple antigens and achieve protection with as few doses as possible.

Ad vectors are known to elicit strong humoral and cell mediated immune responses in IFNAR^-/-^ mice [37]. Therefore, an Ad-based platform was utilized here to address whether IFNAR^-/-^ mice can be protected from lethal CCHFV infection using only the more conserved CCHFV N as the antigen. In this study, IFNAR^-/-^ mice vaccinated with Ad-N developed IgG responses to CCHFV N (S1 Fig) and were partially protected from CCHFV challenge (Figs 1–3; S2 Fig). Due to a lack of GPC antigen in the vaccine preparations protection is unlikely to be mediated by neutralizing antibodies but rather due to priming of CD4+ and CD8+ T-cells and/or non-neutralizing antibody responses as has been previously reported for the Ad platform in other IFNAR^-/-^ vaccine studies [38]. Additional experiments are required to demonstrate which of the CD4+, CD8+ T cell and/or antibody responses after vaccination/infection are responsible for protection. Several studies have evaluated correlates of protection in IFNAR^-/-^ mice to CCHFV infection. The role of adaptive immune responses following MVA immunization was evaluated using the transfer of antibodies and/or T-cells to naïve IFNAR^-/-^ mice [36], while vaccine studies employing DNA and VLP vaccination were evaluated using circulating cytokine profiling [24] and/or IgG subtype ratios [24, 25]. These or alternate experiments, such as selective depletion of B- and T-cell populations following vaccination, could also determine the importance of each of the two arms of the adaptive immune system in response to Ad vaccination. However, it is important to caution that despite their utility and appropriateness for early stage vaccine testing, IFNAR^-/-^ mice are severely immuno-compromised, inbred mice and therefore may generate different protective immune responses than immuno-competent outbred populations of humans and/or animals [12].

While DNA and MVA platforms have demonstrated the protective potential of GPC, vaccine studies utilizing N as the sole antigen are rare and have been non-protective [20, 21, 24, 25]. Thus, protection by Ad-N as demonstrated here provides critical information for future development of CCHF vaccines and suggests that Ad-N protects by mechanisms distinct, and perhaps complimentary, to that of the MVA and DNA platforms. The identification of a protective Ad-based vaccine could therefore be critical in developing an effective CCHFV combination vaccine regimen, in addition to uncovering other immune targets and mechanisms of protection.

Due to the zoonotic nature of CCHF and the limited potential for person-to-person transmission, mass-scale vaccinations are unlikely to be used in case of CCHF. However, as has been recently reported for Ebola, having an efficacious vaccine on hand for emergencies can help to contain outbreaks and protect medical staff attending patients [39]. To this end having readily available vaccine stockpiles which can be efficiently produced, have an acceptable shelf-life, can be efficiently delivered to patients and clinical staff in outbreak regions, and have undergone at least clinical phase II testing would be of great importance against sporadic, life-threatening, emerging infectious diseases such as CCHF. The Ad platform fulfils these criteria and should, in addition to other vaccines, be considered for vaccine stockpiles.

In conclusion, here we report partial protective efficacy of an Ad-based vaccine vector expressing the CCHFV N (Ad-N) against lethal CCHFV challenge in a highly susceptible, immunocompromised mouse model. Partial efficacy of up to 78% following a prime-boost vaccination strategy mediated by this more conserved CCHFV antigen demonstrates its critical role in protection and suggest its future consideration for CCHFV vaccine strategies.

## Supporting information

S1 Fig Antigen expression and immunogenicity. (A) 293 cells were infected with Ad-N or Ad-wt (MOI = 5). Two days post infection, 293 cells were harvested, lysed in SDS lysis buffer and analyzed by SDS-PAGE and immunoblotting. Expression of CCHFV N was demonstrated utilizing rabbit N1028 polyclonal antiserum. (B) IFNAR^-/-^ mice (n = 3) were immunized with Ad-N (prime-boost regimen) and antibody responses (IgG) were detected by ELISA four weeks after boost vaccination. Lysed whole CCHFV particles derived from infected SW13 cells and supernatant from mock-infected SW13 cells were used as positive and negative antigens, respectively. The serum of naïve IFNAR^-/-^ mice (n = 3) was used as a negative control (naïve) and serum from IFNAR^-/-^ mice (n = 2), which had survived a CCHFV infection after vaccination, was used as a positive control (survivor). A 2-fold serum dilution range (1:50–1:6400) was used with the cut-off for a positive dilution set at >3 standard deviations above the reading of negative samples.
(PPTX)

S2 Fig Spleen histopathology and CCHFV antigen distribution in single-dose and prime-boost vaccinated and challenged mice. Groups of IFNAR^-/-^ mice were either single-dose (1.25×10^7^ IFU; intramuscular) or prime-boost (1.25×10^7^ IFU; intramuscular / 10^8^ IFU; intranasal) vaccinated with Ad-N or Ad-wt and challenged with 1000 LD_50_ of CCHFV 28 days following final vaccination. Mice (n = 9 per group) were anesthetized, bled and euthanized to harvest organ samples on day 3 post CCHFV challenge. Thin-sections of spleen material were stained with hematoxylin and eosin (H&E) or with N1028 rabbit polyclonal serum (anti-CCHFV N serum) (IHC). (A) Spleen H&E of control-vaccinated mice (Ad-wt), (B) Spleen H&E of prime-vaccinated mice (Ad-N); (C) Spleen H&E of prime-boost-vaccinated mice (Ad-N); (D) Spleen IHC of control-vaccinated mice (Ad-wt); (E) Spleen IHC of prime-vaccinated mice (Ad-N); (F) Spleen IHC of prime-boost-vaccinated mice (Ad-N). Images are at a magnification of 10x with 500x insets.
(PPTX)
