# Supplementary figures and images for: Nucleocapsid protein-based vaccine provides protection in mice against lethal Crimean-Congo hemorrhagic fever virus challenge
Source: PLoS Negl Trop Dis. 2018 Jul 16;12(7):e0006628. doi: 10.1371/journal.pntd.0006628 (PMC6062107; doi:10.1371/journal.pntd.0006628)

## Slide 1
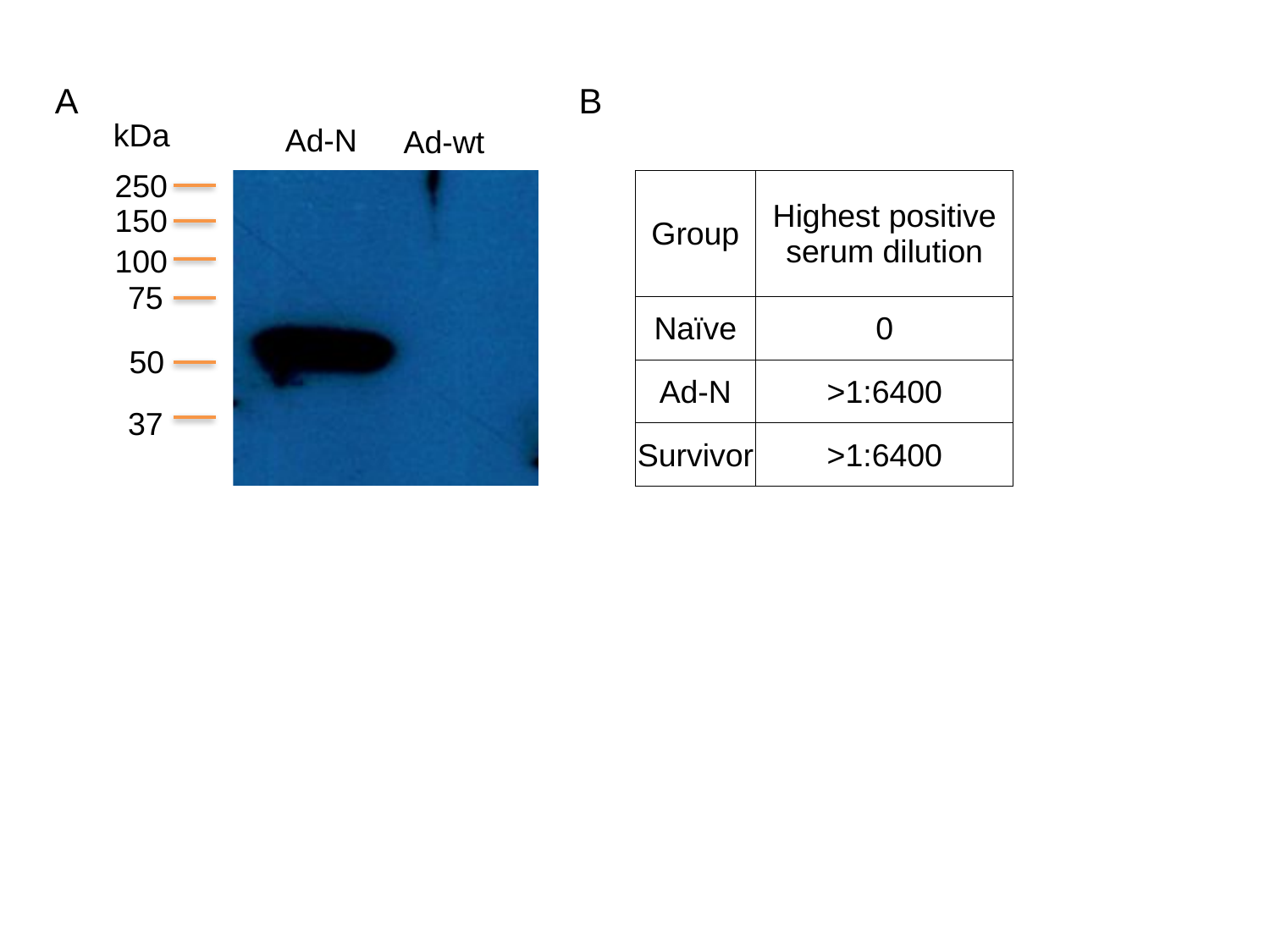

A
B
kDa
Ad-N
Ad-wt
250
| Group | Highest positive serum dilution |
| --- | --- |
| Naïve | 0 |
| Ad-N | >1:6400 |
| Survivor | >1:6400 |
150
100
75
50
37

Supplement: S1 Fig — (A) 293 cells were infected with Ad-N or Ad-wt (MOI = 5). Two days post infection, 293 cells were harvested, lysed in SDS lysis buffer and analyzed by SDS-PAGE and immunoblotting. Expression of CCHFV N was demonstrated utilizing rabbit N1028 polyclonal antiserum. (B) IFNAR-/- mice (n = 3) were immunized with Ad-N (prime-boost regimen) and antibody responses (IgG) were detected by ELISA four weeks after boost vaccination. Lysed whole CCHFV particles derived from infected SW13 cells and supernatant from mock-infected SW13 cells were used as positive and negative antigens, respectively. The serum of naïve IFNAR-/- mice (n = 3) was used as a negative control (naïve) and serum from IFNAR-/- mice (n = 2), which had survived a CCHFV infection after vaccination, was used as a positive control (survivor). A 2-fold serum dilution range (1:50–1:6400) was used with the cut-off for a positive dilution set at >3 standard deviations above the reading of negative samples. (PPTX) [file pntd.0006628.s001.pptx]

## Slide 1
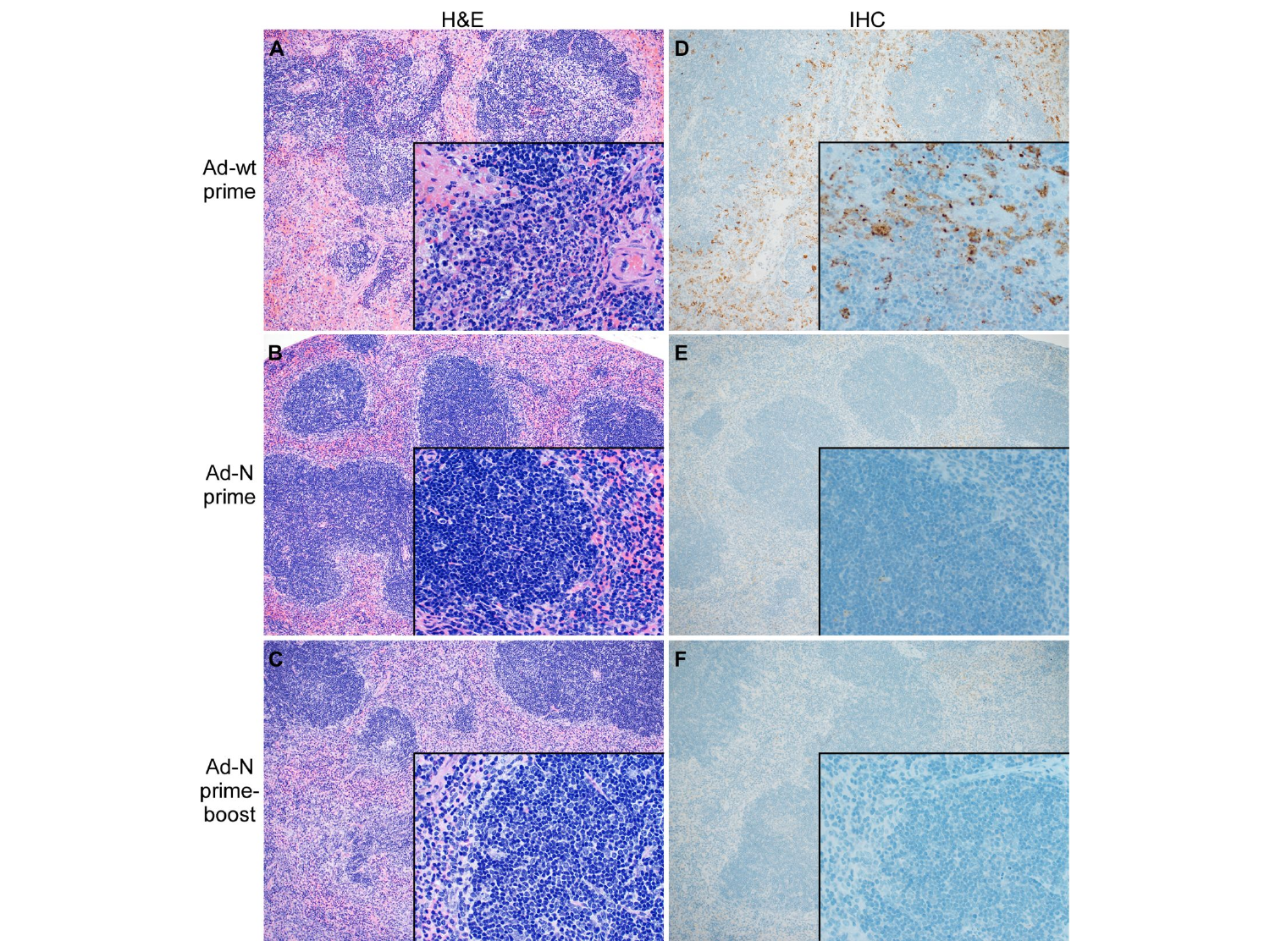

Supplement: S2 Fig — Groups of IFNAR-/- mice were either single-dose (1.25×10^7^ IFU; intramuscular) or prime-boost (1.25×10^7^ IFU; intramuscular / 108 IFU; intranasal) vaccinated with Ad-N or Ad-wt and challenged with 1000 LD50 of CCHFV 28 days following final vaccination. Mice (n = 9 per group) were anesthetized, bled and euthanized to harvest organ samples on day 3 post CCHFV challenge. Thin-sections of spleen material were stained with hematoxylin and eosin (H&E) or with N1028 rabbit polyclonal serum (anti-CCHFV N serum) (IHC). (A) Spleen H&E of control-vaccinated mice (Ad-wt), (B) Spleen H&E of prime-vaccinated mice (Ad-N); (C) Spleen H&E of prime-boost-vaccinated mice (Ad-N); (D) Spleen IHC of control-vaccinated mice (Ad-wt); (E) Spleen IHC of prime-vaccinated mice (Ad-N); (F) Spleen IHC of prime-boost-vaccinated mice (Ad-N). Images are at a magnification of 10x with 500x insets. (PPTX) [file pntd.0006628.s002.pptx]
